# Shh Signaling from the Nucleus Pulposus Is Required for the Postnatal Growth and Differentiation of the Mouse Intervertebral Disc

**DOI:** 10.1371/journal.pone.0035944

**Published:** 2012-04-27

**Authors:** Chitra Lekha Dahia, Eric Mahoney, Christopher Wylie

**Affiliations:** 1 Division of Orthopaedic Surgery, Cincinnati Children's Hospital Medical Center, Cincinnati, Ohio, United States of America; 2 Division of Developmental Biology, Cincinnati Children's Hospital Medical Center, Cincinnati, Ohio, United States of America; Leibniz Institute for Age Research – Fritz Lipmann Institute (FLI), Germany

## Abstract

Intervertebral discs (IVD) are essential components of the vertebral column. They maintain separation, and provide shock absorbing buffers, between adjacent vertebrae, while also allowing movements between them. Each IVD consists of a central semi-liquid nucleus pulposus (NP) surrounded by a multi-layered fibrocartilagenous annulus fibrosus (AF). Although the IVDs grow and differentiate after birth along with the vertebral column, little is known about the mechanism of this. Understanding the signals that control normal IVD growth and differentiation would also provide potential therapies for degenerative disc disease, which is the major cause of lower back pain and affects a large proportion of the population. In this work, we show that during postnatal growth of the mouse, Sonic hedgehog (Shh) signaling from the NP cells controls many aspects of growth and differentiation of both the NP cells themselves and of the surrounding AF, and that it acts, at least partly, by regulating other signaling pathways in the NP and AF. Recent studies have shown that the NP cells arise from the embryonic notochord, which acts as a major signaling center in the embryo. This work shows that this notochord-derived tissue continues to carry out a major signaling function in the postnatal body and that the IVDs are signaling centers, in addition to their already known functions in the mechanics of vertebral column function.

## Introduction

Intervertebral discs (IVDs) allow movement and resistance to tension and compression forces between each vertebrae, and maintain a constant intervertebral space that prevents compression of the spinal nerves. Each disc consists of an annulus fibrosus (AF); a series of orthogonally arranged fibrocartilagenous layers passing between adjacent vertebrae, surrounding a more cellular central area, the nucleus pulposus (NP) containing large reticular cells embedded in a dense aqueous matrix of proteoglycans and collagens [1], [2]. Between the NP and growth plate of the adjacent vertebral body is a layer of tissue several cell layers thick, which form a mineralized layer of cartilage known as the end plate (EP) during the first few weeks of postnatal life in the mouse [3].

Damaged IVDs are a major cause of lower back pain in humans, and of days lost from work [4]–[6]. Their surgical repair is complex, expensive, prone to failure, and does not cure the underlying pathogenesis [5]. Despite its clinical importance, the intervertebral disc is a somewhat neglected structure. Billions of dollars are spent each year on its repair, and yet we know surprisingly little about the mechanisms of its growth, differentiation, and maintenance, nor how these are affected by aging or acute injury [5].

We have shown previously that many intercellular signaling pathways are active during postnatal IVD growth in the mouse [7]. Elucidation of the functions of these would offer new opportunities for biological therapies for both acute and chronic disorders of the IVD. For example, sonic hedgehog (Shh) is synthesized in abundance by the NP cells [3], [7]–[10], consistent with their derivation from the embryonic notochord [8]. In addition to its structural role in the embryo, the notochord is known to be a major signaling center that controls differentiation of the adjacent spinal cord and somites. We have shown previously that during early postnatal growth and differentiation of the mouse IVD, cells of the disc respond to Hedgehog (Hh) signaling [7]. This signaling becomes inactive a few weeks after birth, suggesting that it is primarily required for IVD growth and/or differentiation.

These data raised the interesting hypothesis that the NP in each disc is a local signaling center that controls the growth and/or differentiation of the IVD. We tested this hypothesis in two ways. First, we cultured mouse lumbar IVD's from postnatal day 4 (P4), a stage at which the IVD is growing and differentiating, and when Shh is being synthesized at high levels by the NP cells [3], [7], [9]. Discs cultured for up to five days in the absence of serum maintained their differentiated state in culture, suggesting that endogenous signals are sufficient for this. When cultured in the presence of cyclopamine, a specific inhibitor of Hh signaling [11]–[14], cell proliferation in the NP stopped, and cell differentiation of NP, AF, and EP was inhibited, as shown by the loss of molecular markers of IVD differentiation. All these effects were rescued by replacing cyclopamine with recombinant Shh (rShh) in the culture medium. Second, we carried out a conditional targeting of Shh during the early postnatal stages, to provide in vivo confirmation of the results seen in cultured discs. The results show that in vitro cultures of the IVD offer a rapid assay for the functions of specific signals during the postnatal stages. They also show that Shh signaling is required in the IVD for many components of its postnatal growth and differentiation.

## Results

### The normal early postnatal disc

**Figure 1:**
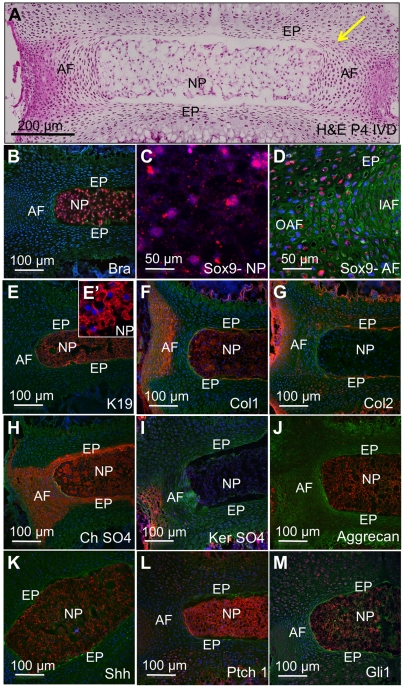
The normal early postnatal disc. The normal structure of the postnatal day four (P4) lumbar IVD are shown. All sections are mid-coronal. (A) shows the general structure of the disc, stained with H&E. B-M show the distribution of molecular markers stained with specific antibodies (red in each case): Brachyury in cell nuclei of the NP (B), Sox 9 in cell nuclei of the NP (C), AF and EP (D), Keratin 19 in the cytoplasm of NP cells (E and E′), collagen 1 (Col1) in NP and outer part of the AF (F), collagen 2 (Col2) in the superficial region of the EP and outer AF (G), chondroitin sulfate (Ch SO4) in both the NP, AF and EP (H), keratin sulfate (Ker SO4) in the outer AF (I), aggrecan in the NP only (J), Shh in the NP only (K), patched-1 (L) and Gli1 (M) in many regions of the IVD and growth plate. Scale bars indicate magnifications used. Green  =  wheat germ agglutinin, blue  =  nuclei stained with POPO-3, NP =  nucleus pulposus, AF =  annulus fibrosus, IAF =  inner AF, OAF =  outer AF, EP =  endplate. For details, see text.

First, we characterized the expression at P4 of several markers of NP, AF, and EP differentiation, since we wish to experimentally manipulate Shh signaling at this stage. (Fig. 1A) shows the histological structure of a normal P4 mouse lumbar IVD (all sections are mid-coronal unless otherwise noted), as well as differentiation markers used in this study. The NP contains large well spread cells, whilst the AF cells are undergoing polarization into layers, and collagens are being laid down in the outermost region [stained pink with eosin in (Fig 1A)]. The AF is continuous with the EP where it meets the growth plate of the vertebra [arrowed in (Fig 1A)]. The T box transcription factor Brachyury (Bra) is expressed only in the NP (Fig. 1B). The HMG box transcription factor Sox9 is expressed by NP, AF and EP cells (Fig. 1C and D). Cytokeratin 19 (K19) is expressed only by the NP cells (Fig. 1E and E′). Collagen 1 is expressed at this stage in the NP, and the outer regions of the AF (Fig. 1F), whilst collagen 2 is expressed in the cells lining the EP and outer regions of the AF (Fig. 1G). Chondroitin sulfate is expressed by all components of the IVD (Fig. 1H), whilst keratan sulfate is expressed in the outer layers of the AF (Fig. 1I). Aggrecan is expressed only in the NP at this stage (Fig. 1J). Shh is expressed at high levels in the NP only (Fig. 1K), whilst the receptor Ptch1 and the downstream target Gli1 are expressed to different extents in all components of the disc (Fig. 1L and M). These data show that there exist good spatially localized cellular and extracellular markers of disc differentiation during the first week after birth that can be used to assay the roles of specific signaling pathways.

### The role of Shh signaling in the postnatal IVD

**Figure 2:**
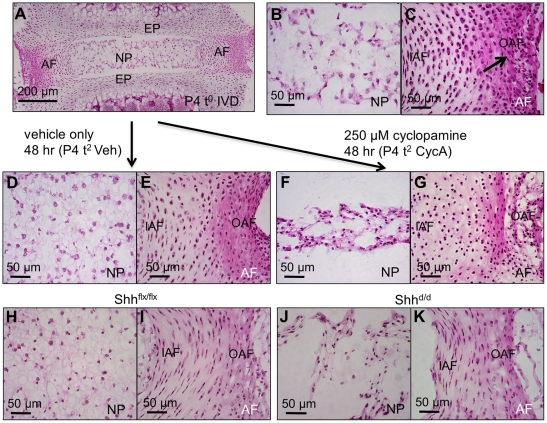
The effects of Shh blockade in P4 cultured discs and of Cre-mediated Shh targeting at P5. (A – C) show the whole disc (A), the NP (B), and AF (C) respectively of a normal disc immediately after explantation (P4 t^0^). (D–G) show the NP and AF respectively after 2 days culture in vehicle only (D and E), or 250 μM cyclopamine (F and G). (H–K) show the NP and AF respectively 5 days after doxycycline treatment at P5 of Shh^flx/flx^; R26rtTA mice (H and I) or of Shh^flx/flx^; (tetO)7-Cre; R26rtTA mice resulting in Cre-mediated targeting of Shh in (J and K). Both cyclopamine in vitro and Shh targeting in vivo have similar effects: the aggregation of NP cells into the center of the disc (F and J) and loss of polarity of the AF cells (G and K). Scale bars indicate magnifications used. NP  =  nucleus pulposus, AF  =  annulus fibrosus (dense peripheral eosin positive collagen layers are arrowed in C), IAF =  inner AF, OAF =  outer AF, EP =  endplate.

Two series of experiments were carried out. First, we established a simple in vitro assay for function by excising single lumbar IVDs at P4, and placing them in organ culture conditions. At this stage, the IVDs are small [<1 mm across, see (Fig. 1A)] and are avascular in vivo, and so nutrients, oxygen, and carbon dioxide, are transported in and out of the disc by diffusion in vivo. (Fig. 2A–C) show the histology of an IVD fixed immediately after removal (P4 t^0^). The NP cells are spread to form a reticular network that fills the disc cavity (Fig. 2B), and the AF cells are already forming concentric layers as shown by the polarized and elongated cell nuclei (Fig. 2C). Dense eosin staining at the periphery of the AF [arrowed in (Fig. 2C)] indicates the laying down of collagens between the AF cells. (Fig. 2D and E) show that the normal histological structures of both the NP and AF are retained in culture for two days (P4 t^2^). In fact, normal histology is maintained for at least five days [see later section, (Figs. 7 and 8)]. Since the discs were cultured without serum, all intercellular signaling required for maintenance of the differentiated structure of the disc up to P4 must be intrinsic to the IVD. Since the NP expresses high levels of Shh, we blockaded Hh signaling in vitro using cyclopamine. (Figs. 2F and G) show sections through the NP and AF regions respectively of IVD's maintained for two days in 250 μM cyclopamine. This was the lowest dose of cyclopamine that completely inhibited Gli1 expression by the NP cells (Figure S1). There were dramatic changes to the histological structures of both the NP and the AF, compared to the control discs (incubated in vehicle only) (Fig. 2D and E). The NP cells lost their characteristic reticular network and collapsed into the center of the disc space (Fig. 2F), and the AF cells lost their obvious polarity and layered structure (Fig. 2G).

To confirm these results in vivo, and to show that the effects of cyclopamine in vitro were due to the specific blockade of Shh secreted by the NP cells, and not due to blockade of Ihh secreted by the hypertrophic zone of the adjacent vertebral growth plates (which were also present in the cultures), we generated mice carrying floxed alleles of Shh, doxycycline-inducible Cre transgene (Shh^flx/flx^; (tetO)7-cre; R26rtTA) as well as mice carrying Cre transgene only. Doxycycline was administered on P5, and the IVD's harvested on P10. NP and AF from doxycycline-treated Shh^flx/flx^; R26rtTA mutant mice which served as controls (Fig. 2H and I), and the data from doxycycline treated Cre control mice were identical (Figure S2). The IVDs had the normal reticular structure of the NP cells (Fig. 2H), and the layered, polarized structure of the AF (Fig. 2I). However, IVDs taken five days after doxycycline treatment from Shh^flx/flx^;(tetO)7-cre; R26rtTA compound mutant mice resulting in deletion of Shh showed a histology (Fig. 2J and K) very similar to that caused by cyclopamine in vitro. The NP cells were collapsed into the center of the disc cavity, and the AF cells lost their polarity and characteristic layered structure. These data suggest that Shh secreted by the NP cells is required for the maintenance of normal differentiation of both NP and AF cells during early life.

### Loss of Shh signaling results in loss of differentiation markers of NP cells

**Figure 3:**
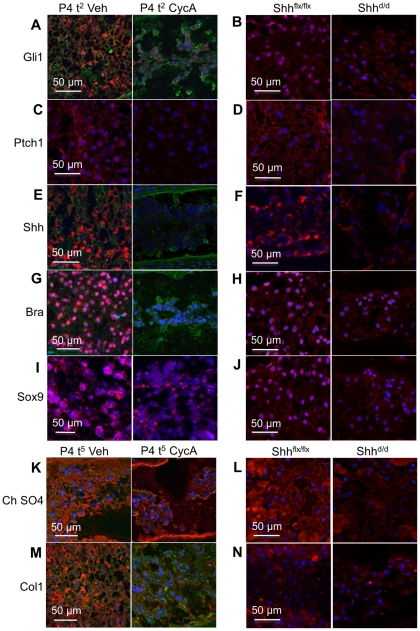
The effects of Shh blockade in vitro and Shh targeting in vivo of molecular markers of NP differentiation. The panels are arranged as in a table. Each vertical column shows a treatment (two or five day vehicle treated control [P4 t^2 or 5^ Veh] and two or five days in cyclopamine [P4 t^2 or 5^ CycA] from the in vitro experiments; and control Shh flox [P5 d^5^ Shh^flx/flx^; R26rtTA] and Shh mutant [P5 d^5^; Shh^d/d^(tetO)7-Cre; R26rtTA] mice from the in vivo experiments). Each horizontal row shows the expression, by immunocytochemistry, of Gli 1 (A) and (B), Ptch1 (C) and (D), Shh (E) and (F), Brachyury (G) and (H), Sox9 (I) and (J), chondroitin sulfate (K) and (L), and collagen 1 (M) and (N). All the above markers show reduced expression after treatment with cyclopamine in vitro, or targeting of Shh in vivo. Scale bars indicate magnifications used. Blue  =  nuclei stained with POPO-3, green  =  counterstain with wheat germ agglutinin. See text for details.

**Figure 4:**
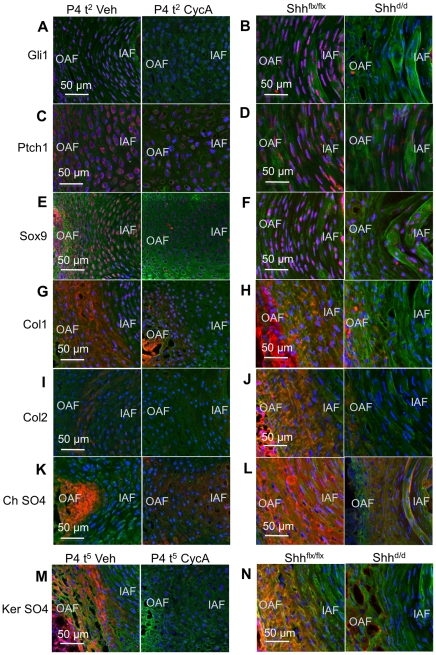
The effects of Shh targeting in vitro and in vivo on the expression of molecular markers in the AF. The panels are arranged as in a table. Each vertical column shows a treatment (two or five day vehicle treated control [P4 t^2 or 5^ Veh] and two or five days in cyclopamine [P4 t^2 or 5^ CycA] from the in vitro experiments; and control Shh flox [P5 d^5^ Shh^flx/flx^; R26rtTA] and Shh mutant [P5 d^5^; Shh^d/d^(tetO)7-Cre; R26rtTA] mice from the in vivo experiments). The rows shows the expression, by immunocytochemistry, or Gli1 (A) and (B), Ptch1 (C) and (D), Sox9 (E) and (F), collagen 1 (G) and (H), collagen 2 (I) and (J), chondroitin sulfate (K) and (L), and keratin sulfate (M) and (N). The expression of all these markers was reduced after treatment with cyclopamine in vitro, or Shh targeting in vivo. Scale bars indicate magnifications used. IAF  =  inner annulus fibrosus, OAF  =  outer annulus fibrosus. Blue  =  cell nuclei stained with POPO-3, green  =  general counterstain with wheat germ agglutinin. See text for details.

**Figure 5:**
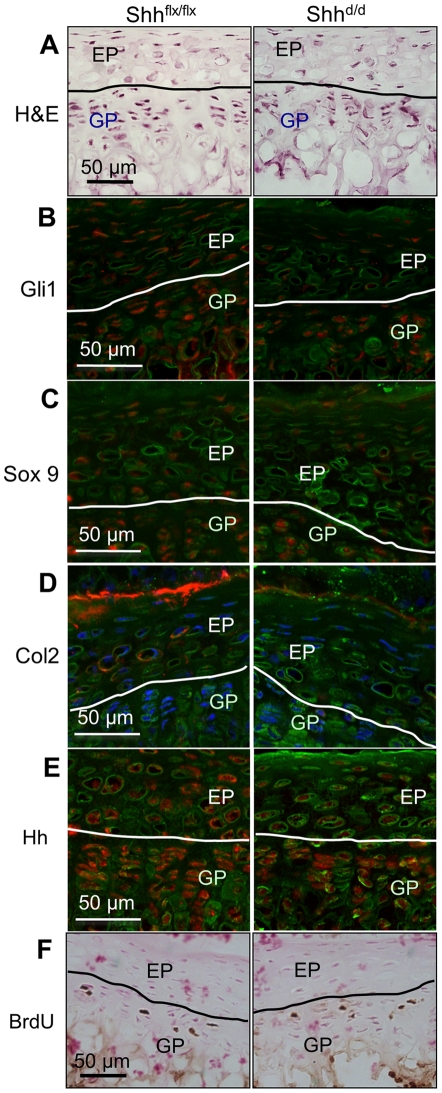
The effects of deletion of Shh signaling on the end plate of the IVDs in vivo. (A) shows H&E staining and histology of EP following doxycycline treatment of the control [P5 d^5^ Shh^flx/flx^; R26rtTA] and Shh mutant mice [P5 d^5^; Shh^d/d^(tetO)7-Cre; R26rtTA]. (B)–(D) shows reduced expression of Gli1, Sox9 and collagen 2 expression in the EP cells and the inner layer of AF following Shh deletion in vivo. (E) shows that Ihh continues to be expressed in the growth plate chondrocytes of the Shh mutant as well as in the control mice, while hedgehog (Hh) expression was undetectable in the EP cells. (F) shows that there is no effect on BrdU incorporation (Brown  =  BrdU positive cells, cell nuclei stained with nuclear fast red) in the growth plate following Shh deletion, compared to the control mice. Scale bars indicate magnifications used. Red  =  expression of the specific protein, Blue  =  cell nuclei stained with POPO-3, green  =  general counterstain with wheat germ agglutinin. EP =  end plate, GP =  growth plate. See text for details.

Histological sections were stained with differentiation markers of NP (Fig. 3), AF (Fig. 4), and EP (Fig. 5). First, to confirm that Shh signaling was blocked by the two treatments, we stained for Shh, Gli1, and Ptch1, which are all known targets of Shh signaling [15]–[19]. Gli1, Ptch1, and Shh expression in the NP cells was dramatically reduced within two days (P4 t^2^) following cyclopamine treatment in vitro (Fig. 3A, C and E), and five days after doxycycline treatment in vivo (Fig. 3B, D and F), showing that Shh signaling was blocked. Shh expression was reduced after conditional deletion in vivo (Fig. 3F), but not as completely as by cyclopamine treatment in vitro (Fig. 3E), suggesting that Cre-mediated excision of the Shh gene was not complete, or that some perduring Shh protein was still present. Next, we analyzed the effects of Shh blockade in vitro and in vivo on the expression of the transcription factors Bra and Sox9 (Fig. 3G–J). Expression of Bra was reduced to levels that were undetectable using the same staining and imaging protocols as the controls following cyclopamine treatment in vitro (Fig. 3G), and reduced but still detectable after in vivo Shh targeting (Fig. 3H). Expression of Sox9 showed the same pattern (Fig. 3I and J). Extracellular matrix glycoproteins, GAGs, and proteoglycans were also reduced in the NP by both treatments (Fig. 3K–N). However, this took longer; so that a much larger reduction in staining intensity was seen after five days in culture [P4 t^5^, (Fig. 3K and M)]. These results show that the loss of histological structure of NP cells coincides with the changes in gene expression.

### Loss of Shh signaling affects differentiation markers of AF cells

The expression of AF differentiation markers was also reduced by Hh blockade in vitro and Shh targeting in vivo (Fig. 4). Gli1 and Ptch1 expression were both reduced after two days in vitro and five days in vivo, showing that the treatments were effective. Once again, the reduction was greatest in vitro (Fig. 4A–D). The AF does not express Bra, but the expression of Sox9 was reduced by both treatments (Fig. 4E and F). Expression of the glycoproteins collagen 1 (Fig. 4G and H) and collagen 2 (Fig. 4I and J) were reduced, as was the expression of the two proteoglycans, chondroitin sulfate (Fig. 4K and L) and keratan sulfate (Fig. 4M and N). These data suggest that AF differentiation is also regulated either directly or indirectly by Shh from NP cells.

### Loss of Shh signaling affects differentiation markers of EP cells

The EP is continuous around the NP with the AF, and so can be considered part of the IVD, but is also immediately adjacent to the proliferative zone of the growth plate, which is known to be controlled by Ihh released from the hypertrophic zone. Thus EP cells could respond to either Shh from the NP, or Ihh from the growth plate, or both. To study this, we assayed the expression of differentiation markers in the EPs after conditional targeting of Shh in vivo. In these specimens, Ihh from the growth plate is still present, but Shh from the NP will be reduced or absent. No major histological differences can been seen in EP of the control versus Shh deleted mice (Fig. 5A). However, Gli1 expression was reduced in the EP, but not in the growth plate chondrocytes, of these animals (Fig. 5B) suggesting that activation of Hh downstream targets in the EP at this stage is at least partially due to Shh from the NP. Sox9 expression was also reduced in the EP cells following loss of Shh, whilst the growth plate chondrocytes showed normal levels of Sox9 expression (Fig. 5C). Collagen 2 expression was lost in the EP cells following loss of Shh signaling (Fig. 5D). This suggests that for these components of their differentiation, the EP cells are controlled by Shh signals from the NP, rather than Ihh signals from the growth plate. Ihh expression is maintained in the growth plates of the Shh-targeted mice (Fig. 5E), and continues to control cell proliferation in the growth plate, as shown by BrdU incorporation (Fig. 5F). This data suggests that the EP cell differentiation is at least partially under the control Shh secreted by the NP cells. It has been shown previously [20] that the EP is affected by targeting of Ihh in the growth plate. It is therefore likely that the EP is controlled by both Hh ligands, as would be expected by its position between the two sources of signaling.

### Absence of the Ihh-secreting hypertrophic zone of the growth plate in vitro does not affect NP cell differentiation in vitro

**Figure 6:**
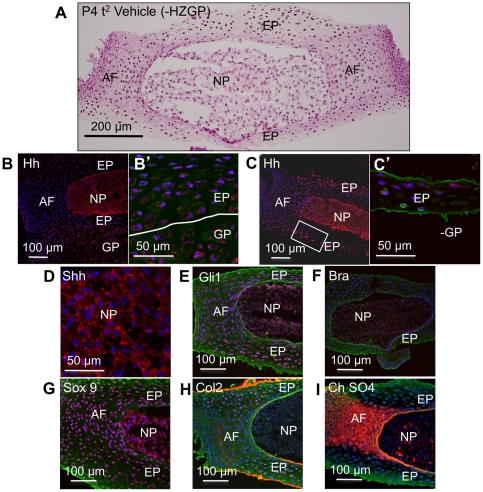
The effects of removal of the hypertrophic zone of the vertebral growth plates (-HZGP) from IVDs before culture. (A) shows their histology after two days in culture. (B–B′) shows low and high magnification immunostaining for both Shh and Ihh, using an antibody that stains both, in the growth plate chondrocytes (GP) and EP before culture. (C–C′) shows low and high magnification images respectively of 48 hour cultures to show that Hh staining is still seen in the EP in the absence of the growth plate. (D–I) show normal levels of expression of Shh, Gli1, Bra, Sox9, collagen 2, and chondroitin sulfate, in discs cultured for 48 hour in the absence of the growth plate. Scale bars indicate magnifications used. Red  =  expression of the specific protein, Blue  =  cell nuclei stained with POPO-3, green  =  general counterstain with wheat germ agglutinin. NP  =  nucleus pulposus, AF  =  annulus fibrosus, EP  =  end plate, GP  =  growth plate. See text for details.

Cultured IVD's contained attached cells from the adjacent growth plates. The hypertrophic zone of the growth plate is known to be a source of Ihh, and cyclopamine treatment of cultured IVD's will thus inhibit both Shh from the NP and Ihh from the growth plate. The in vivo targeting specifically of Shh had the same effects as those of cyclopamine in culture, suggesting that the effects seen in the cultured IVD are specifically due to Shh inhibition. However, in an attempt to dissect the functions of the two Hh's in vitro, we carefully dissected the hypertrophic zones from isolated P4 IVD's before culture. After culture for two days, the histological structure of the disc was normal (Fig. 6A). Immunostaining using an antibody against both Shh and Ihh showed the presence of both ligands in the whole IVD before dissection (Fig. 6B and B′), and the presence of Hh staining in all components of the IVD after removal of the hyptertrophic zone and culture for 2 days (Fig. 6C and C′). The expression of Gli1, and the differentiation markers Bra, Sox9, collagen 2, and chondroitin sulfate continue to be at normal levels after culture for 2 days in the absence of the growth plate (Fig. 6D–I). Similar pattern of histology and expression levels of differentiation markers were observed in the P4 IVDs cultured for five day (P4 t^5^), from which the hypertrophic zone was dissected prior to culture (Figure S3). This data supports the conclusion from in vivo targeting of Shh that IVD's are responding to Shh secreted by the NP. Of course we cannot exclude the possibility that some aspects of IVD differentiation are controlled by Ihh, since not all differentiation markers were assayed.

### Recombinant Shh rescues the cyclopamine phenotype in vitro

**Figure 7:**
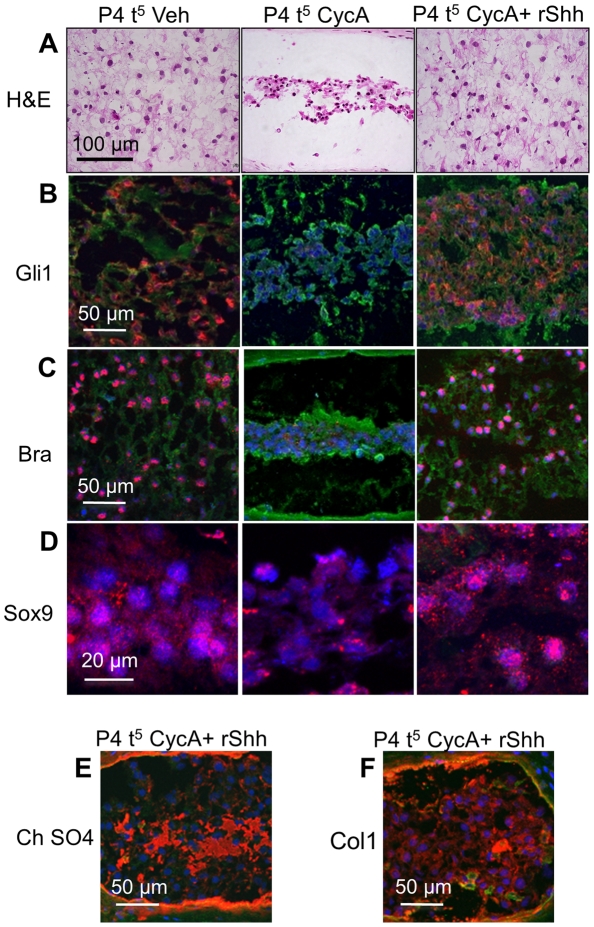
The effects of cyclopamine phenotype in NP cells can be rescued and are reversible in vitro. The panels are arranged as a table. Each row shows the staining pattern, and each vertical column shows the treatment (P4 t^5^ Veh  =  five day vehicle treated control, P4 t^5^ CycA  =  five days in cyclopamine, P4 t^5^ CycA+ rShh  =  two days in cyclopamine followed by three days in rShh. H&E staining (A) shows collapsed NP cells are reversed after replacing cyclopamine with rShh. (B)–(D) show that the downstream targets of Shh, Gli1, Bra, and Sox9 are all reversed. (E) and (F) show that expression of chondrotin sulfate, and collagen 1 is also reversed. The vehicle treated and cyclopamine treated samples for similar time points have previously shown in (Figure 3K) (chondroitin sulfate) and 3M (collagen 1). Scale bars indicate magnifications used. Red  =  expression of the specific protein, Blue  =  cell nuclei stained with POPO-3, green  =  general counterstain with wheat germ agglutinin. See text for details.

**Figure 8:**
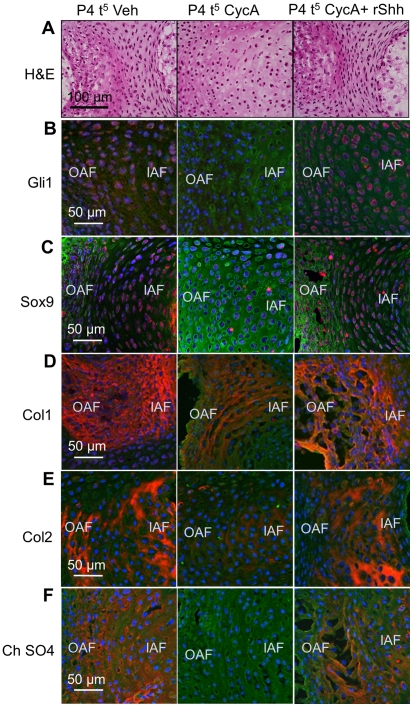
The molecular markers of the AF are also reversed by rShh after cyclopamine treatment. Each row shows the staining pattern, and each column shows the treatment. P4 t^5^ Veh  =  five day vehicle treated control, P4 t^5^ CycA  =  five days in cyclopamine, P4 t^5^ CycA+ rShh  =  two days in cyclopamine followed by three days in rShh. (A) shows by H&E staining that loss of AF cell polarity is reversed by replacement of cyclopamine with rShh. (B)–(F) shows that expression of Gli1, Sox9, collagen 1, collagen 2, and chondroitin sulfate, respectively, are also reversed. Scale bars indicate magnifications used. IAF =  inner annulus fibrosus, OAF =  outer annulus fibrosus. Red  =  expression of the specific protein, Blue  =  cell nuclei stained with POPO-3, green  =  general counterstain with wheat germ agglutinin. See text for details.

As an additional control for non-Hh off-target effects of cyclopamine in vitro, we washed out cyclopamine after two days culture, and replaced it with 100 ng/mL recombinant Shh (rShh) for three further days (P4 t^5^). The effects of Shh blockade on the NP were reversed by this treatment, including the reticular structure of the NP cells (Fig. 7A), expression of Gli1 (Fig. 7B) and Bra (Fig. 7C), Sox9 (Fig. 7D) chondroitin sulfate (Fig. 7E, compare with Fig. 3K), and collagen 1 [(Fig. 7F), compare with (Fig. 3M)]. Addition of recombinant Shh also reversed the effects of cyclopamine on the AF, including the histological structure (Fig. 8A), expression of Gli1 (Fig. 8B), Sox9 (Fig. 8C), collagen 1 (Fig. 8D), collagen 2 (Fig. 8E) and chondroitin sulfate (Fig. 8F). These data show that the observed effects were due to loss of Hh signaling, rather than non-specific effects of the cyclopamine treatment. Cultures from which cyclopamine was removed, but not replaced by Shh [(Figure S4); P4 t^2^ CycA t^3–5^ Veh], did not recover significantly within the three day period.

### Shh signaling is required for postnatal proliferation of NP cells

**Figure 9:**
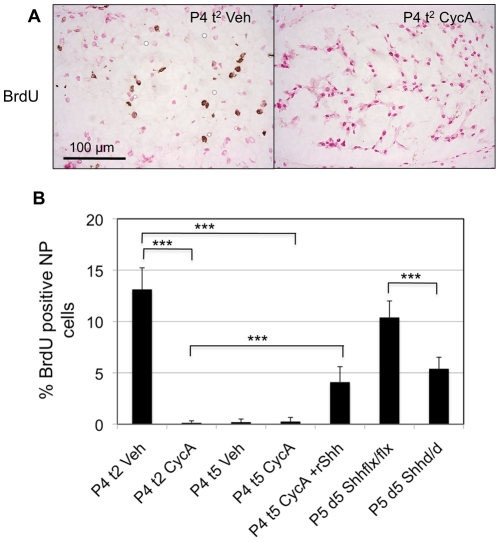
Shh signaling is required for proliferation of NP cells. Figure 9 shows that cyclopamine treatment in vitro, and targeting of Shh in vivo both cause loss of NP cell proliferation. (A) shows representative images of BrdU staining of NP from IVDs cultured for two days in vehicle (P4 t^2^ Veh) and cyclopamine (P4 t^2^ CycA) treated medium. The percentage of BrdU-positive NP cells from different experimental groups in the study is quantified in (B) which shows decrease in the percentage of BrdU positive NP cells from both the in vitro and in vivo experiments. The percentage of BrdU positive cells increased in the NP cells from rShh rescue experiment carried out in vitro. Graph shows the s.d. of the mean for each data point. Significance was calculated using unpaired student's t-test, and *** represents p≤ 0.001. Brown  =  BrdU positive cells, cell nuclei stained with nuclear fast red.

To test the role of hedgehog signaling on cell proliferation discs cultured for two or five days with or without cyclopamine were treated with bromo-deoxyuridine (BrdU) 24 hours before being snap-frozen, sectioned, fixed and stained for incorporated BrdU. Many NP cells were BrdU-positive in vehicle-treated control IVDs after two days, but not after five days, in culture (Fig. 9A and B). In vivo, cell proliferation continues until P14–21 [3]. The discrepancy between in vitro and in vivo data suggests that continued cell proliferation in the NP requires continued systemic signals from the circulation. Few, if any labeled cells were found in the AF or EP (data not shown). (Fig. 9A) shows an example of the effect of cyclopamine after two-day culture compared to a vehicle-treated control disc. No BrdU incorporation in the NP was seen after cyclopamine treatment. (Fig. 9B) shows the percentage of BrdU-positive NP cells from the different experimental conditions, assayed by expressing the number of BrdU-positive cells as a percentage of the total number of nuclei in each section. A total of twelve sections were analyzed from four discs for each treatment. The error bars represent the standard deviation to show the variation in different experimental samples. The p-value was calculated between two groups to compare the effects of Shh blockade or rescue using the unpaired student's t-test. BrdU incorporation was significantly (p≤0.001) inhibited by incubation in cyclopamine, indicating that Shh signaling controls, either directly or indirectly, the proliferation of the NP cells. Replacing cyclopamine with rShh, followed by three days of further culture, caused significant increase (p≤0.001) in the cell proliferation of NP compared to the NP cells from both the P4 t^2^ vehicle treated as well as P4 t^2^ cyclopamine treated IVDs. However, cell proliferation was not rescued back to control levels by Shh replacement in vitro. BrdU incorporation was also inhibited in the in vivo experiments using the Shh mutant mice [see P5 d^5^ Shh flx/flx compared with P5 d^5^ Shh d/d bars in (Fig. 9B), p≤0.001]. However, the effect was not as great as seen by cyclopamine treatment in vitro [see P4 t^2^ Veh compared with P4 t^2^ CycA bars in (Fig. 9B)]. This could be due to the fact that some Shh protein remains after five days of doxycycline treatment [see (Fig. 3F)]. It could also be due to some level of control by Ihh from the growth plate in vivo. Double in vivo targeting would be required to resolve this point.

### Shh is the key regulator of other major cell signaling pathways

**Figure 10:**
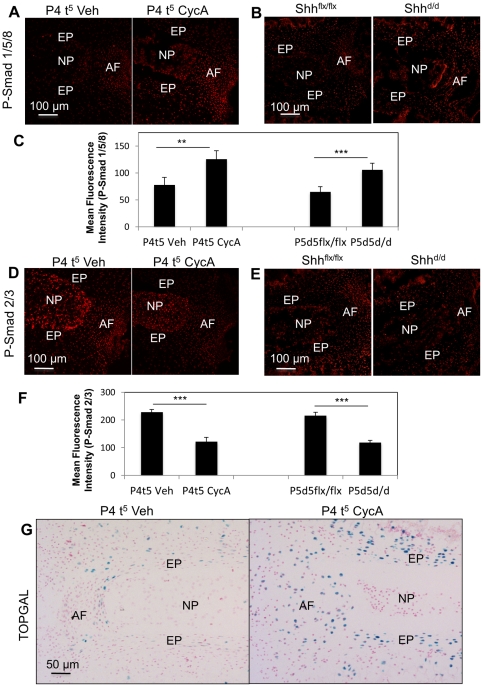
Shh is the key regulator of other major cell signaling pathways. Figure 10 shows that cyclopamine treatment in vitro, and targeting of Shh in vivo both cause alterations in activities of other signaling pathways. (A) and (B) show increases in BMP signaling and (C) shows the graph for the intensity of immunostaining from both the experiments. (D) and (E) show decreases in TGFβ signaling represented by graph plotted for the intensity of immunostanining (E). (F) shows an increase in canonical Wnt signaling. In (A)–(D), red  =  expression of the specific protein. In E, blue  =  β-gal positive cells, pink  =  nuclei stained with nuclear fast red.

Blockade of Shh signaling in vitro, or targeting in vivo, had a large range of effects on the IVD. This suggests that Shh may either act directly on a large number of downstream targets, or may act indirectly by controlling other signaling pathways. To distinguish between these possibilities, we assayed for the levels of activity of different signaling pathways in IVD's treated with cyclopamine or from in vivo targeting studies, and compared these with treatment controls. For BMP signaling we used an antibody against the phosphorylated forms of Smad 1, 5, and 8 [21]. BMP signaling was increased in all components of IVD following both in vivo and in vitro loss of Shh signaling (Fig. 10A–C). For TGFβ signaling, we used an antibody against the phosphorylated forms of Smad 2, 3 [22]. TGFβ signaling was reduced in IVD cells following both in vitro and in vivo blockade of Shh signaling (Fig. 10D–F).

To analyze the affects of loss of Shh signaling on canonical Wnt signaling pathway in vitro, IVDs from TOPGAL reporter mice were cultured under the same conditions and duration as described above, either in vehicle only, or cyclopamine, for five days (P4 t^5^). We consistently see a reduction in TOPGAL expression during culture (not shown), suggesting that canonical Wnt signaling requires systemic signaling, which will be absent in culture. However, when Hh signaling was blockaded, Wnt signaling was upregulated in both AF and EP cells (Fig. 10G). These data suggest that Shh signaling regulates other major cell signaling pathways in the IVD during active growth and differentiation.

## Discussion

**Figure 11:**
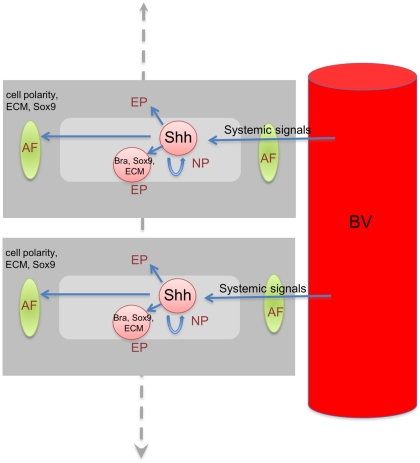
Postnatal regulation of IVD. Schema to show how systemic circulating signals control and coordinate the growth and differentiation of all the IVDs by controlling expression of local signals such as Shh. Two IVDs are shown for simplicity. The dotted line indicates the same events throughout the vertebral column. BV  =  blood vessel.

The data presented here show that cell signaling intrinsic to the IVD controls both cell proliferation in the NP, and the expression of disc differentiation markers in the IVD. The culture periods were not systematically extended to see how long normal disc structure could be maintained in the absence of exogenous signals. However, it was interesting to note that, although disc differentiation was maintained in culture, it did not progress as it would have done in vivo. Discs cultured for five days maintained the structure they had at P4 t^0^, rather than attaining the structure and size they would have reached by P9. In addition, cell proliferation in the NP's of cultured discs did not continue past five days in vitro (the equivalent of P9 in vivo), but normally extends to P14 before ceasing in vivo [3]. These data suggest that signaling pathways within the disc are in turn controlled by signals from the circulation to control the continued progression of disc growth and differentiation. (Fig. 11) shows a schema of this proposed mechanism. There is also a possibility that the reduced growth of the IVD in longer duration of culture can be due to lack of nutritional factors in the culture medium.

Second, Shh is a major signaling pathway controlling IVD growth and differentiation. Shh has been shown previously by our lab and others to be synthesized in the NP [3], [7]–[10]. Shh is also importnat for the formation of the notochord sheath and patterning of embryonic nucleus pulposus cells in the embryo [23]. However, neither its role, nor the extent of its importance in IVD postnatal development have been previously appreciated. A particularly interesting finding was that NP cells signal to each other (or themselves). Many of the effects of cyclopamine are on the NP cells that synthesize the Shh. Interestingly, cyclopamine treatment resulted in loss of Shh expression in the NP cells, suggesting that a feedback loop controls Shh protein expression in the disc. Although we did not identify all the functions of Shh in the IVD, nor all of its targets, we found that these include the maintenance of differentiation markers in both the NP and the AF, as well as cell proliferation in the NP. In the future it will be important to identify all the targets of Shh signaling.

Control of the EP is likely to be particularly interesting, as it is sandwiched between cells releasing Shh (the NP) and Ihh (the growth plate). In this study, we found that expression of Gli1, Sox9, and collagen 2 were reduced after in vivo targeting of Shh expression at P5. However, previous work has shown that the EP is also affected by Ihh targeting in the growth plate [20]. Based on these combined data, it is likely that the EP is controlled by both signaling sources. Whether this is a quantitative or qualitative difference in signal strength will require further experiments.

Third, the pleiotropic effects of Shh signaling found here seem to be due in part to its regulation of transcription factors, such as Sox9, known to regulate the synthesis of several extracellular matrix components such as collagen 1, collagen 2 and chondroitin sulfate [24]–[26] and in part due to its control of other signaling pathways in the IVD. Blockade of Shh caused the down-regulation of TGFβ signaling, and up-regulation of BMP and Wnt signaling. In the future, it will be important to identify precisely which other pathways are controlled by Shh, how it does this, and how the major signaling pathways intersect to control the holistic postnatal growth and differentiation of the disc. For example, we show here that BMP and Wnt signaling are up-regulated by Shh blockade, and it has been shown that, during avian somite patterning, the BMP inhibitor noggin acts downstream of both Shh and Wnt signaling to inhibit BMP4 activity [27]. It will be interesting to see if the same mechanism is acting in the IVD.

It will also be important in the future to elucidate the links between systemic growth signals and the local signals produced in the disc. It is likely, for example, that Shh expression by the NP cells, or its action on other signaling pathways, or both, are controlled by systemic signals which coordinate all the NP's along the vertebral column so that the discs all grow and differentiate at the same rates and times [exemplified by the two discs shown in (Fig. 11)]. Most importantly, these data show that it may be possible to control both growth and differentiation of diseased discs, by the application of appropriate agonists and antagonists of the signaling pathways found to carry out specific functions in vivo.

## Materials and Methods

### Mice

FVB mice were maintained in accordance with the National Institutes of Health Guide for the Care and Use of Laboratory Animals, and all experiments were carried out in strict accordance with institutional guidelines under Institutional Animal Care and Use Committee (IACUC) approval at Cincinnati Children's Hospital Research Foundation (CCHRF). IACUC at CCHRF approved the study described in this manuscript with Animal Use Protocol number 9A01004. All studies were carried out using at least four IVDs in each group and repeated three times.

### Generation of doxycycline inducible Shh flx/flx compound transgenic mice

Shh flx/flx mice [28], [29] were interbred with (tetO)7-Cre [30] and rosa26 rtTA IRES eGFP [31] mouse lines to obtain Shh flx/flx; (tetO)7-Cre; R26 rtTA compound offspring and genotyped as previously described. The postnatal day five (P5) pups which were intraperitoneally injected with 2 μg of doxycycline (D9891, Sigma-Aldrich) in a final volume of 50 μl, on P5 and P7 and the lumbar vertebrae were collected for analysis on P10. All the pups were administered 50 μl of BrdU 10 hours before sample collection.

### Organ culture of neonatal IVDs

Lumbar vertebrae (LV) were dissected from four-day postnatal mice (P4) and placed in ice-cold 1x phosphate buffered saline (PBS) containing antibiotics (pen/strep). The IVDs were dissected out from the LV along with the adjacent vertebral growth plates under a dissecting microscope. One batch of IVDs was snap frozen at t^0^ as control in vivo samples (P4 t^0^). The IVDs were then placed on type-IV Collagen (354233; BD Biosciences) coated Millicell®-CM cell culture inserts (PICM01250; Millipore) and cultured in 24-well culture plates in DMEM Ham F-12 containing 1% pen/strep and supplemented with 1x Insulin-Transferrin-Sodium Selenite supplement (11074547001; Roche Diagnostics) The culture was maintained at 37°C and 5% CO_2_. To study the effect of loss of Shh signaling, one group of IVDs were treated with 250 μM cyclopamine (C-8700; LC Laboratories). The treatment was carried out for two-five days (t^2^ − t^5^) depending on the culture duration. The vehicle -treated discs served as controls. In order to study the specificity of cyclopamine action on Shh signaling pathway, the IVDs were initially treated with cyclopamine for two days, then the medium was removed and the IVDs were thoroughly washed. The discs were further cultured for another three-days in medium supplemented with 100 ng/mL of recombinant Shh [rmShh(C25II)-N; 464-Sh; Research Diagnostics] or medium containing vehicle only. At the end of the culture the IVDs were washed three times in 1x PBS and snap frozen in OCT^TM^ molds.

### BrdU incoporation assay

Bromo-deoxy-Uridine (BrdU) assays were carried out using Amersham^TM^ Cell Proliferation Kit (RPN20; GE Healthcare) as per manufacture's instructions. BrdU (1∶1000) was added to the culture medium 24 hours before the termination of culture, at which the IVDs were washed three times in 1x PBS and snap frozen in OCT^TM^ molds. Cryosections were collected from the IVDs of the in vitro and in vivo experiments at 6 μm thickness, fixed in 2% paraformaldehyde (PFA) and washed thrice in 1x PBS. Next, the sections were treated with 2% H_2_0_2_ diluted in methanol to quench the endogenous perxoxidase activity, which otherwise would interfere with the interpretation of the results. The sections were washed three times in 1x PBS and incubated with anti-BrdU antibody for 2 hours at room temperature, followed by 30 minutes incubation with HRP-conjugated secondary antibody. To visualize the BrdU positive cells under a microscope, the sections were treated with the DAB Plus Substrate Staining System (Thermo Scientific), and the nuclei were counter-stained pink with nuclear fast red (N3020; Sigma). The sections were dehydrated and mounted in xylene based mounting medium. Sections were photographed using a Zeiss axiovert microscope and NP cells were counted using the Axio vision software.

### Histology

Cryosections of the cultured and t^0^-control IVDs, and from the Shh mutant mice were collected at a thickness of 6 μm in the coronal plane, using a Leica cryostat. Sections were processed immediately, or stored at -20°C for later use. Histology of the vertebrae was analyzed by fixing sections in 2% PFA and staining with hematoxylin and eosin (H&E). Sections were photographed using a Zeiss axiovert microscope.

### Immunolocalization analysis

Immunostaining was carried out on 6 μm thick coronal sections of IVDs from in vitro and in vivo experiments. The sections were fixed in 2% PFA for 2 minutes, permeabilized using 1x PBS containing 0.2% Triton-x100 for 20 minutes, blocked in blocking buffer (10% donkey or goat serum, 4% bovine serum albumin in 1x PBS containing 0.1% Triton-x100 [PBST]) for one hour, treated with diluted primary antibody (Brachyury, sc-17743, Santa Cruz; Sox9, Ab3697, Abcam; Cytokeratin 19, TROMA-III, Hybridoma Bank; Collagen I, GTX41285, Gene Tex®, Inc; Collagen II, NB600-844, Novus Biologicals; Chondroitin sulfate, ab11570, Abcam; Keratan sulfate, 270427-1, Associates of Cape Cod Inc.; Aggrecan, ab3778, Abcam; Shh, S4944, Sigma-Aldrich; Ptch1, MAB41051, R? Gli1, AF3455, R? Ihh, ab52919 (also detects Shh), abcam; phospho Smad 1, 5 and 8, 9511, Cell Signaling; phospho Smad 2 and 3, sc-11769-R, Santa Cruz) overnight at 4°C in a humidified chamber, washed in PBST (three times), treated with 15 μg/mL of the appropriate Cy5-conjugated secondary antibody (Goat anti Mouse; Donkey anti Goat; Goat anti Rabbit; Goat anti Rat, from Jackson ImmunoResearch Laboratories, INC) in blocking buffer for an hour at room temperature, in the dark, and washed in PBS (twice). The sections were counterstained with POPO-3^TM^ iodide (1∶4000, Molecular Probes) and wheat germ agglutinin (1∶1000, Molecular Probes) to stain the cell nuclei and cell membranes respectively, washed in 1x PBS (twice) and mounted in PBS containing 50% glycerol and 25 μg/mL of the anti-quenching agent DABCO^TM^ (D27802, Sigma). Images were captured using a Zeiss LSM 510 confocal microscope. Control sections were incubated in the appropriate secondary antibody only, and were all negative (data not shown).

### X-gal staining

Lumbar vertebrae were collected from P4 TOPGAL mice and the dissected IVDs were cultured as described above. At the end of culture period, the IVDs were embedded and snap frozen in OCT molds. β-galactosidase staining was carried out on 6 μm thick sections collected in the coronal plane following fixation in 2% PFA for two minutes and three washes in 1x PBS. Sections were washed twice for 10 minutes each in 2 mM MgCl_2_, permeabilized using 0.02% NP-40 and 2 mM MgCl_2_, stained for β-gal activity using X-gal in the staining solution (5 mM K_3_Fe(CN)_6_, 5 mM K_4_Fe(CN)_6_, 2 mM MgCl_2_, 0.01% NaDeoxycholate, 0.02% NP-40, 1 mg/mL X-gal made in 1x PBS) at 37°C overnight. After washing twice in PBS the slides were counterstained with nuclear fast red (N3020, Sigma) dehydrated through an ethanol series and mounted. Sections were photographed using a Zeiss Axiovert microscope.

### Statistical analysis

Statistical analysis was carried out by counting the percentage of BrdU-positive NP cells from the total NP cells on individual section of different treatment groups. The statistical analyses of the immunostaining studies was carried out by measuring the mean fluorescence intensities in the entire IVD from individual sections and different treatment groups. The means and s.d. of the values obtained from individual experiments were calculated. Charts show the s.d. of the mean for each data point. Significance was calculated using unpaired student's t-test.

## Supporting Information

Figure S1 **Dose response of cyclopamine on Gli1 expression in P4 cultured IVDs.** P4 IVDs were cultured for five days (P4 t^5^) either in vehicle (A), or in increasing concentrations of cyclopamine (B-E). At the end of the culture cyrosections were collected at 6 μm thickness and stained using a specific antibody for Gli1 (red). (A) shows Gli1 expression in all the regions of the vehicle treated control IVDs. (B) to (E) show progressive reduction of Gli1 expression with increasing doses of cyclopamine (50–250 μM respectively). Scale bars indicate magnifications used. Red  =  expression of Gli1. NP =  nucleus pulposus, AF =  annulus fibrosus, EP =  end plate.
(TIF)

Figure S2 **Doxycycline treatment of tetO-cre transgenic mice shows no change in the phenotype of the NP (A), AF (B), and EP (C) cells.** Immunostaining for differentiation markers used for comparison are as shown in Figures 3, 4 and 5 in the manuscript. (A) Shows that the expression of Gli1, ptch1, Shh, Bra, Sox9, chondroitin sulfate, and collagen 1 continues to be expressed in the NP cells. (B) Shows the expression of Gli1, ptch1, Sox9, collagen 1, collagen 2, chondroitin sulfate and keratin sulfate are present in the AF cells. (C) Shows histology and immunostaining for Gli1, Sox9, collagen 2 and Hh in the EP cells. Scale bars indicate magnifications used. IAF =  inner annulus fibrosus, OAF =  outer annulus fibrosus. Red  =  expression of specific protein, Blue  =  cell nuclei stained with POPO-3, green  =  general counterstain with wheat germ agglutinin. EP  =  end plate, GP  =  growth plate.
(TIF)

Figure S3 Shows that P4 IVDs cultured for five days (P4 t^5^) after removal of the hypertrophic zone of the vertebral growth plates (-HZGP) continue to have normal histology (A) and expression of the differentiation markers (B–G) shown in Figure 6 in the manuscript. (A) Shows the reticular network of NP cells and the layers of the AF cells, and intact EP cells. (B and B′) show immunostanining for Hh protein in the NP and EP cells. (C–G) Show the normal expression of Gli1, Brachyury, Sox9, collagen 2 and chondroitin sulfate in the IVD cultured for five days on the absence of the growth plates on both the adjacent ends. Scale bars indicate magnifications used. Red  =  expression of specific protein, Blue  =  cell nuclei stained with POPO-3, green  =  general counterstain with wheat germ agglutinin. NP  =  nucleus pulposus, AF =  annulus fibrosus, EP  =  end plate.
(TIF)

Figure S4 Shows that P4 IVDs cultured in the presence of cyclopamine for two days followed by culturing in vehicle only medium for another three days (P4 t^2^ CycA t^3–5^ Veh) does not reverse the expression of Gli1 (A), Shh (B), and Sox9 (C) in the NP cells. Scale bars indicate magnifications used. Red  =  expression of specific protein, Blue  =  cell nuclei stained with POPO-3, green  =  general counterstain with wheat germ agglutinin.
(TIF)

## References

[1] Deyo RA, Tsui-Wu YJ (1987). Descriptive epidemiology of low-back pain and its related medical care in the United States.. Spine (Phila Pa 1976).

[2] Pooni JS, Hukins DW, Harris PF, Hilton RC, Davies KE (1986). Comparison of the structure of human intervertebral discs in the cervical, thoracic and lumbar regions of the spine.. Surg Radiol Anat.

[3] Dahia CL, Mahoney EJ, Durrani AA, Wylie C (2009). Postnatal growth, differentiation, and aging of the mouse intervertebral disc.. Spine (Phila Pa 1976).

[4] Frymoyer JW, Wiesel SW, Weinstein JN, Herkowitz HN, Dvorak J, Bell GR (1996). Magnitude of the problem.. The Lumbar Spine.

[5] Ireland D (2009). Molecular mechanisms involved in intervertebral disc degeneration and potential new treatment strategies.. Bioscience Horizons.

[6] Urban JP, Roberts S (2003). Degeneration of the intervertebral disc.. Arthritis Res Ther.

[7] Dahia CL, Mahoney EJ, Durrani AA, Wylie C (2009). Intercellular signaling pathways active during intervertebral disc growth, differentiation, and aging.. Spine (Phila Pa 1976).

[8] Choi KS, Cohn MJ, Harfe BD (2008). Identification of nucleus pulposus precursor cells and notochordal remnants in the mouse: implications for disk degeneration and chordoma formation.. Dev Dyn.

[9] Dahia CL, Mahoney EJ, Durrani AA, Wylie C (2011). Intercellular Signaling Pathways Active During and After Growth and Differentiation of Lumbar Vertebral Growth Plate.. Spine (Phila Pa 1976).

[10] DiPaola CP, Farmer JC, Manova K, Niswander LA (2005). Molecular signaling in intervertebral disk development.. J Orthop Res.

[11] Berman DM, Karhadkar SS, Hallahan AR, Pritchard JI, Eberhart CG (2002). Medulloblastoma growth inhibition by hedgehog pathway blockade.. Science.

[12] Chen JK, Taipale J, Cooper MK, Beachy PA (2002). Inhibition of Hedgehog signaling by direct binding of cyclopamine to Smoothened.. Genes Dev.

[13] Dray N, Tessmar-Raible K, Le Gouar M, Vibert L, Christodoulou F (2010). Hedgehog signaling regulates segment formation in the annelid Platynereis.. Science.

[14] Surace EM, Balaggan KS, Tessitore A, Mussolino C, Cotugno G (2006). Inhibition of ocular neovascularization by hedgehog blockade.. Mol Ther.

[15] Bastida MF, Sheth R, Ros MA (2009). A BMP-Shh negative-feedback loop restricts Shh expression during limb development.. Development.

[16] Capdevila J, Estrada MP, Sanchez-Herrero E, Guerrero I (1994). The Drosophila segment polarity gene patched interacts with decapentaplegic in wing development.. EMBO J.

[17] Lum L, Beachy PA (2004). The Hedgehog response network: sensors, switches, and routers.. Science.

[18] McMahon AP (2000). More surprises in the Hedgehog signaling pathway.. Cell.

[19] Sanz-Ezquerro JJ, Tickle C (2000). Autoregulation of Shh expression and Shh induction of cell death suggest a mechanism for modulating polarising activity during chick limb development.. Development.

[20] Maeda Y, Nakamura E, Nguyen MT, Suva LJ, Swain FL (2007). Indian Hedgehog produced by postnatal chondrocytes is essential for maintaining a growth plate and trabecular bone.. Proc Natl Acad Sci U S A.

[21] Faure S, de Santa Barbara P, Roberts DJ, Whitman M (2002). Endogenous patterns of BMP signaling during early chick development.. Dev Biol.

[22] Faure E, Heisterkamp N, Groffen J, Kaartinen V (2000). Differential expression of TGF-beta isoforms during postlactational mammary gland involution.. Cell Tissue Res.

[23] Choi KS, Harfe BD (2011). Hedgehog signaling is required for formation of the notochord sheath and patterning of nuclei pulposi within the intervertebral discs.. Proc Natl Acad Sci U S A.

[24] Bell DM, Leung KK, Wheatley SC, Ng LJ, Zhou S (1997). SOX9 directly regulates the type-II collagen gene.. Nat Genet.

[25] Lefebvre V, Huang W, Harley VR, Goodfellow PN, de Crombrugghe B (1997). SOX9 is a potent activator of the chondrocyte-specific enhancer of the pro alpha1(II) collagen gene.. Mol Cell Biol.

[26] Tew SR, Pothacharoen P, Katopodi T, Hardingham TE (2008). SOX9 transduction increases chondroitin sulfate synthesis in cultured human articular chondrocytes without altering glycosyltransferase and sulfotransferase transcription.. Biochem J.

[27] Hirsinger E, Duprez D, Jouve C, Malapert P, Cooke J (1997). Noggin acts downstream of Wnt and Sonic Hedgehog to antagonize BMP4 in avian somite patterning.. Development.

[28] Dassule HR, Lewis P, Bei M, Maas R, McMahon AP (2000). Sonic hedgehog regulates growth and morphogenesis of the tooth.. Development.

[29] Lewis PM, Dunn MP, McMahon JA, Logan M, Martin JF (2001). Cholesterol modification of sonic hedgehog is required for long-range signaling activity and effective modulation of signaling by Ptc1.. Cell.

[30] Perl AK, Wert SE, Nagy A, Lobe CG, Whitsett JA (2002). Early restriction of peripheral and proximal cell lineages during formation of the lung.. Proc Natl Acad Sci U S A.

[31] Belteki G, Haigh J, Kabacs N, Haigh K, Sison K (2005). Conditional and inducible transgene expression in mice through the combinatorial use of Cre-mediated recombination and tetracycline induction.. Nucleic Acids Res.

