# Supplementary figures and images for: Shh Signaling from the Nucleus Pulposus Is Required for the Postnatal Growth and Differentiation of the Mouse Intervertebral Disc
Source: PLoS One. 2012 Apr 27;7(4):e35944. doi: 10.1371/journal.pone.0035944 (PMC3338762; doi:10.1371/journal.pone.0035944)

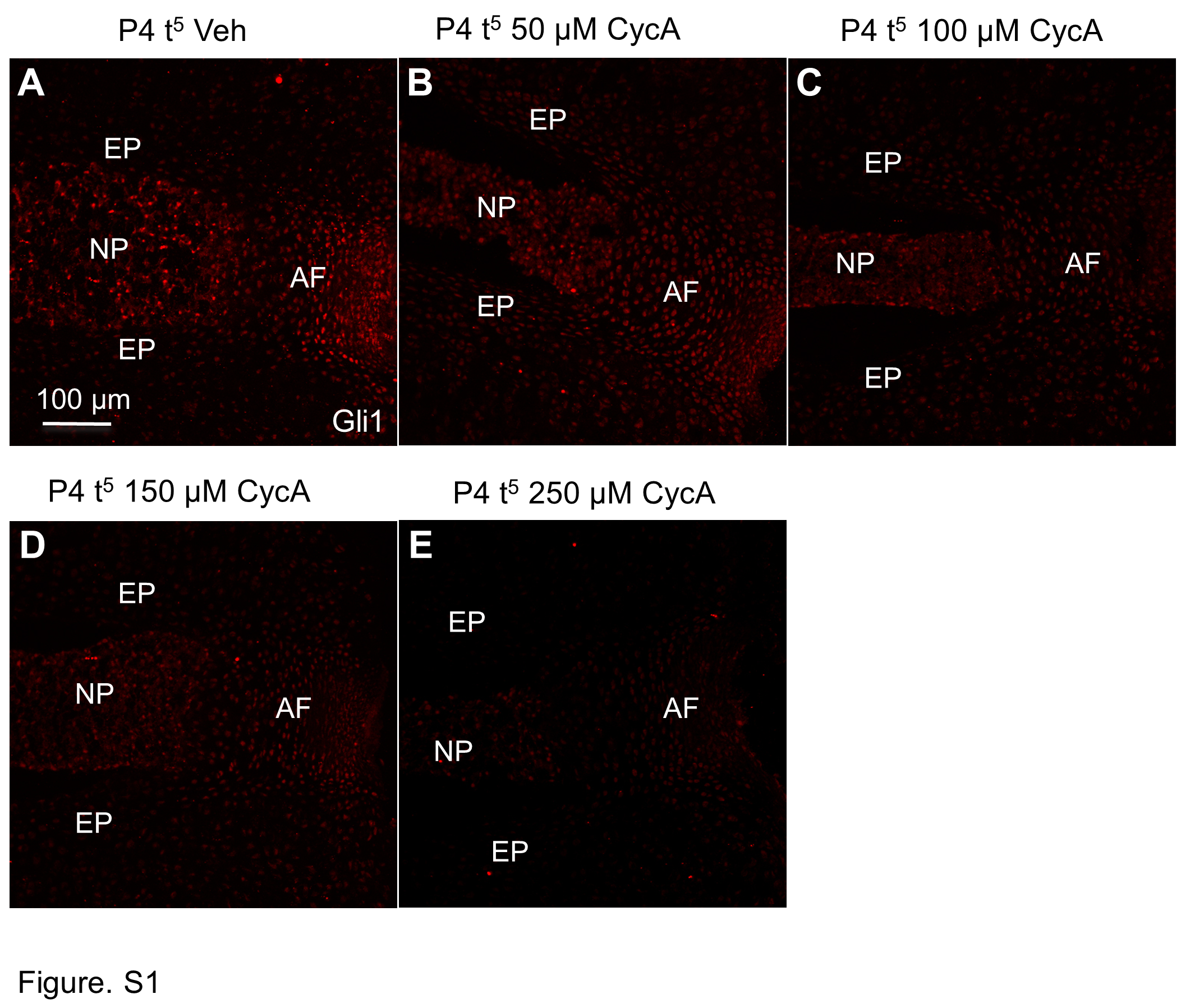

Supplement: Figure S1 — Dose response of cyclopamine on Gli1 expression in P4 cultured IVDs. P4 IVDs were cultured for five days (P4 t5) either in vehicle (A), or in increasing concentrations of cyclopamine (B-E). At the end of the culture cyrosections were collected at 6 μm thickness and stained using a specific antibody for Gli1 (red). (A) shows Gli1 expression in all the regions of the vehicle treated control IVDs. (B) to (E) show progressive reduction of Gli1 expression with increasing doses of cyclopamine (50–250 μM respectively). Scale bars indicate magnifications used. Red = expression of Gli1. NP = nucleus pulposus, AF = annulus fibrosus, EP = end plate. (TIF) [file pone.0035944.s001.tif]

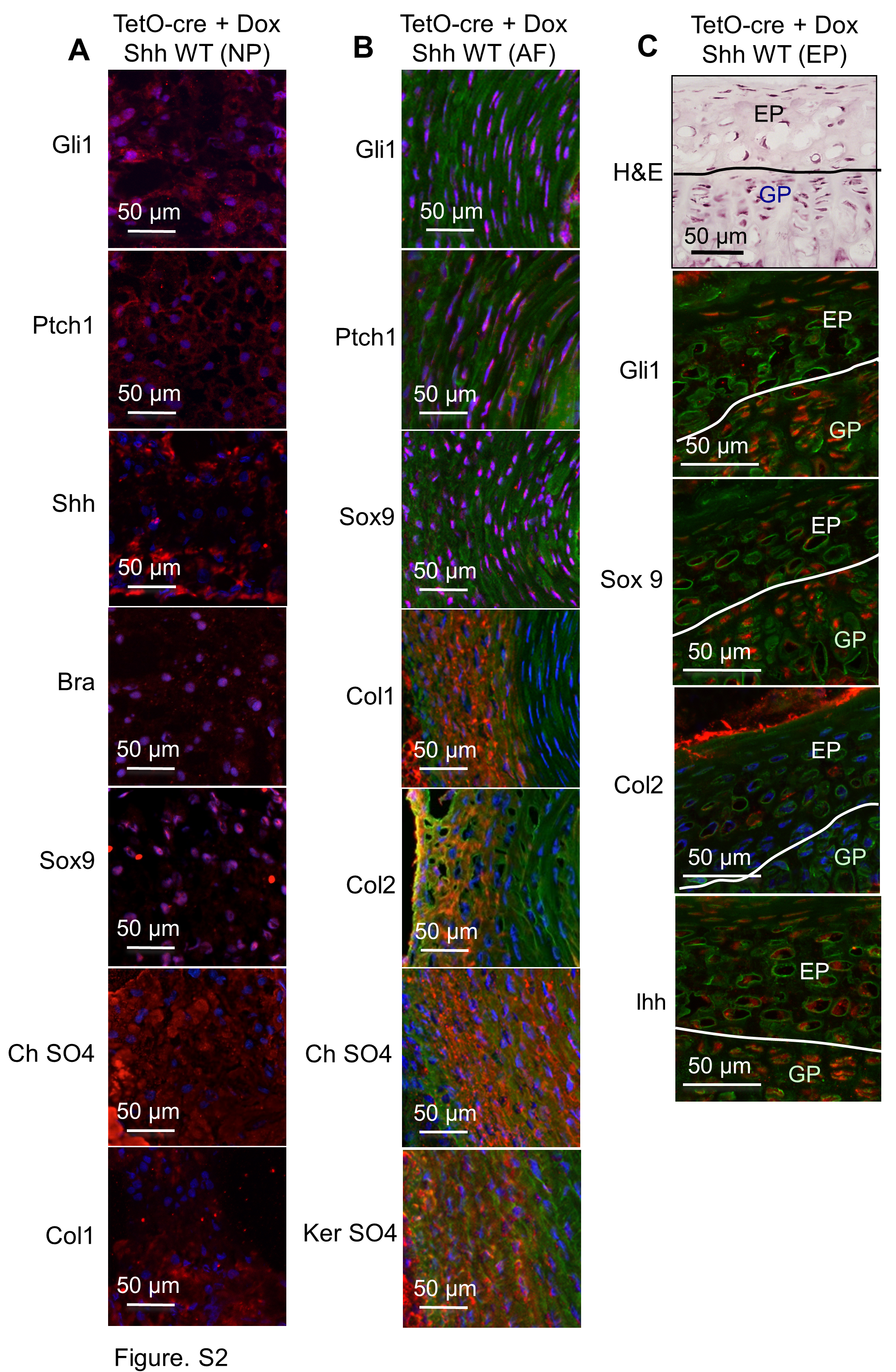

Supplement: Figure S2 — Doxycycline treatment of tetO-cre transgenic mice shows no change in the phenotype of the NP (A), AF (B), and EP (C) cells. Immunostaining for differentiation markers used for comparison are as shown in Figures 3, 4 and 5 in the manuscript. (A) Shows that the expression of Gli1, ptch1, Shh, Bra, Sox9, chondroitin sulfate, and collagen 1 continues to be expressed in the NP cells. (B) Shows the expression of Gli1, ptch1, Sox9, collagen 1, collagen 2, chondroitin sulfate and keratin sulfate are present in the AF cells. (C) Shows histology and immunostaining for Gli1, Sox9, collagen 2 and Hh in the EP cells. Scale bars indicate magnifications used. IAF = inner annulus fibrosus, OAF = outer annulus fibrosus. Red = expression of specific protein, Blue = cell nuclei stained with POPO-3, green = general counterstain with wheat germ agglutinin. EP = end plate, GP = growth plate. (TIF) [file pone.0035944.s002.tif]

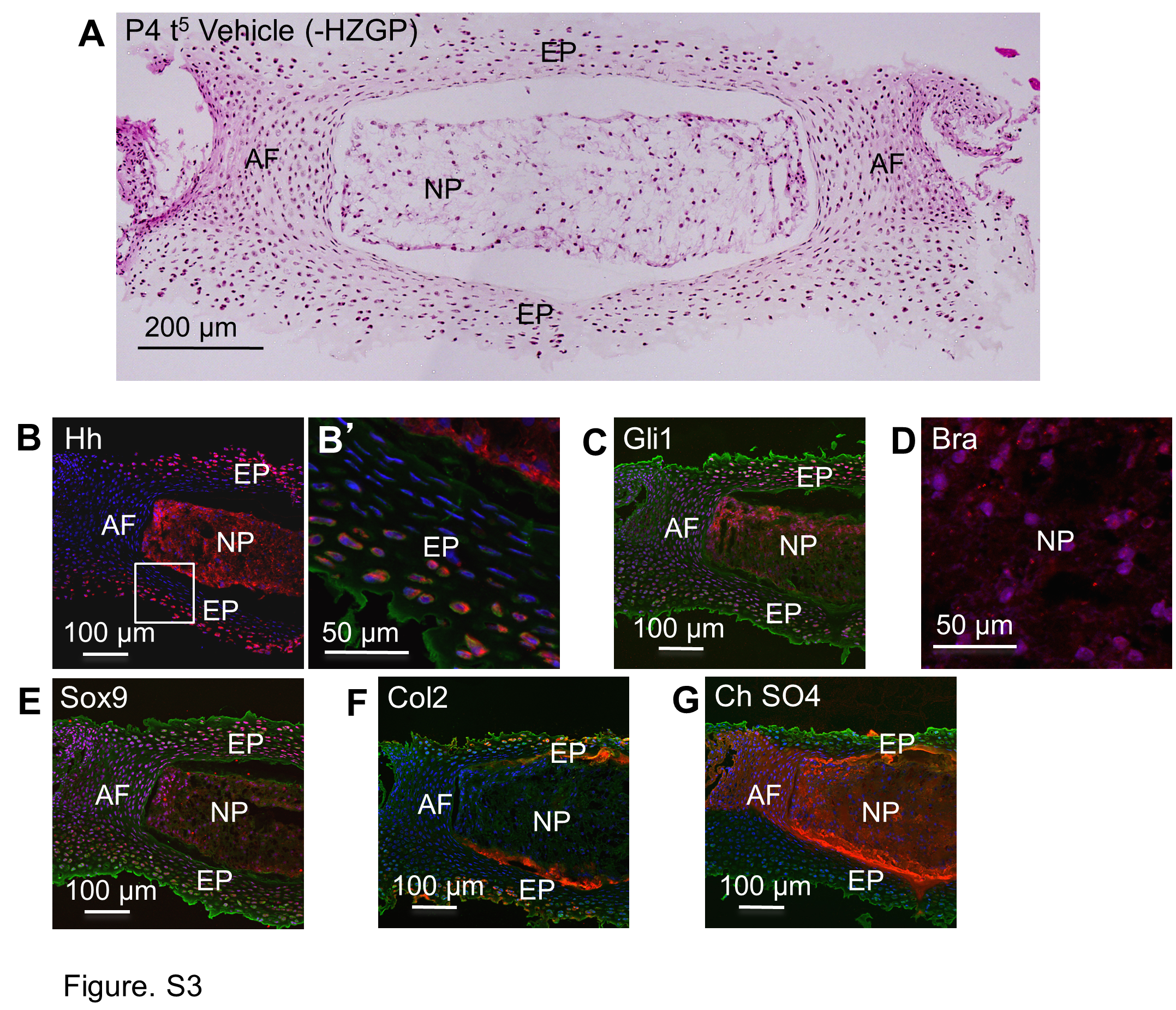

Supplement: Figure S3 — Shows that P4 IVDs cultured for five days (P4 t5) after removal of the hypertrophic zone of the vertebral growth plates (-HZGP) continue to have normal histology (A) and expression of the differentiation markers (B–G) shown in Figure 6 in the manuscript. (A) Shows the reticular network of NP cells and the layers of the AF cells, and intact EP cells. (B and B′) show immunostanining for Hh protein in the NP and EP cells. (C–G) Show the normal expression of Gli1, Brachyury, Sox9, collagen 2 and chondroitin sulfate in the IVD cultured for five days on the absence of the growth plates on both the adjacent ends. Scale bars indicate magnifications used. Red = expression of specific protein, Blue = cell nuclei stained with POPO-3, green = general counterstain with wheat germ agglutinin. NP = nucleus pulposus, AF = annulus fibrosus, EP = end plate. (TIF) [file pone.0035944.s003.tif]

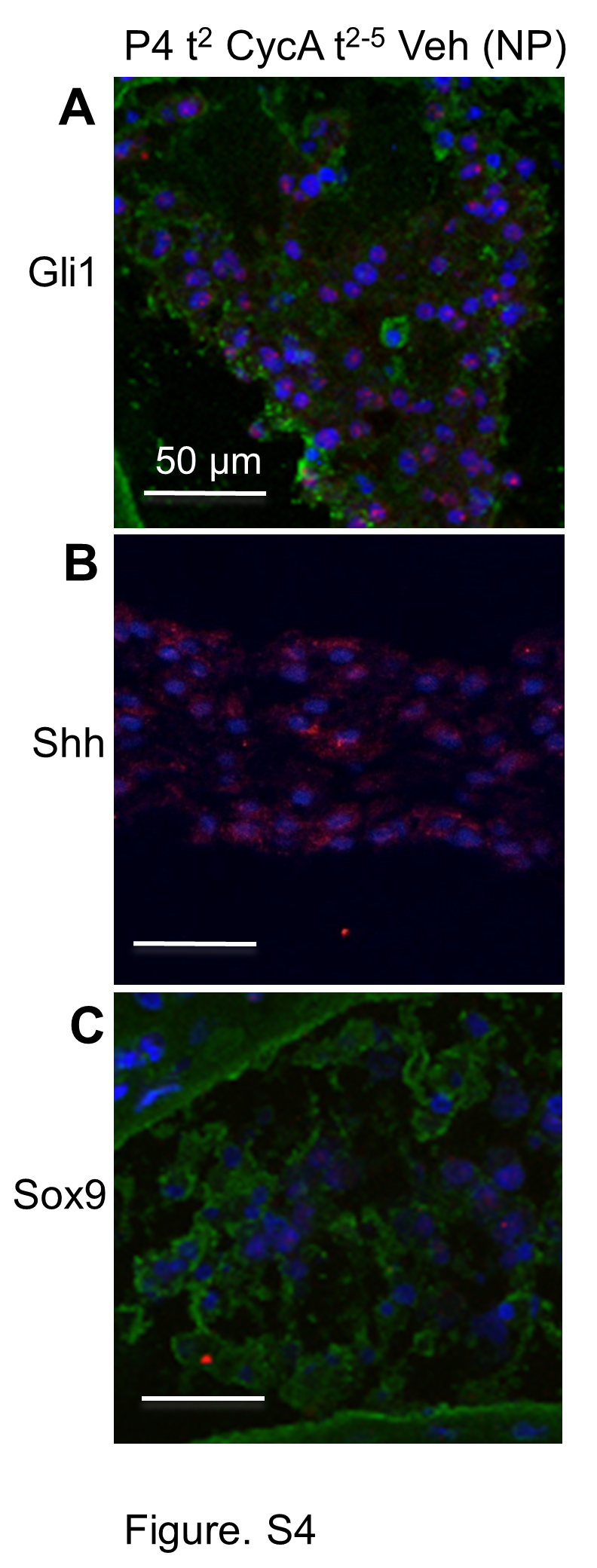

Supplement: Figure S4 — Shows that P4 IVDs cultured in the presence of cyclopamine for two days followed by culturing in vehicle only medium for another three days (P4 t2 CycA t3–5 Veh) does not reverse the expression of Gli1 (A), Shh (B), and Sox9 (C) in the NP cells. Scale bars indicate magnifications used. Red = expression of specific protein, Blue = cell nuclei stained with POPO-3, green = general counterstain with wheat germ agglutinin. (TIF) [file pone.0035944.s004.tif]
